# Cell Signaling through Protein Kinase C Oxidation and Activation

**DOI:** 10.3390/ijms130910697

**Published:** 2012-08-24

**Authors:** Daniela Cosentino-Gomes, Nathália Rocco-Machado, José Roberto Meyer-Fernandes

**Affiliations:** 1 Institute of Medical Biochemistry, Federal University of Rio de Janeiro (UFRJ), CCS, Bloco H, Cidade Universitária, Ilha do Fundão, 21941-590, Rio de Janeiro, RJ, Brazil; E-Mails: rocco@bioqmed.ufrj.br (N.R.-M.); meyer@bioqmed.ufrj.br (J.R.M.-F.); 2 Institute of National Science and Technology of Structural Biology and Bioimage (INCTBEB), CCS, Bloco H, Cidade Universitária, Ilha do Fundão, 21941-590, Rio de Janeiro, RJ, Brazil

**Keywords:** protein kinase C, reactive oxygen species, cell signaling

## Abstract

Due to the growing importance of cellular signaling mediated by reactive oxygen species (ROS), proteins that are reversibly modulated by these reactant molecules are of high interest. In this context, protein kinases and phosphatases, which act coordinately in the regulation of signal transduction through the phosphorylation and dephosphorylation of target proteins, have been described to be key elements in ROS-mediated signaling events. The major mechanism by which these proteins may be modified by oxidation involves the presence of key redox-sensitive cysteine residues. Protein kinase C (PKC) is involved in a variety of cellular signaling pathways. These proteins have been shown to contain a unique structural feature that is susceptible to oxidative modification. A large number of scientific studies have highlighted the importance of ROS as a second messenger in numerous cellular processes, including cell proliferation, gene expression, adhesion, differentiation, senescence, and apoptosis. In this context, the goal of this review is to discuss the mechanisms by which PKCs are modulated by ROS and how these processes are involved in the cellular response.

## 1. Introduction

Phosphorylation, acetylation, ubiquitinylation, and glycosylation are among the most well-known post-translational modifications. The concept of protein oxidation as a post-translational modification has only recently gained acceptance. Protein oxidation occurs as an outcome of a chemical attack by reactive oxygen species (ROS) or reactive nitrogen species (RNS) on susceptible amino acids, such as tyrosine, tryptophan, histidine, lysine, methionine, and cysteine [1]. Indeed, it is important to note that signal transduction must occur in a coordinated manner in response to a stimulus. The key elements of a signaling response are reversibility and specificity [2]. In this way, an oxidation-dependent chain reaction may be short and employ only a low concentration of oxidants to avoid irreversible damage to cellular components. Thus, proteins that are reversibly modulated by these reactant molecules are of high interest. Kinases and phosphatases, which act coordinately in the regulation of signal transduction through the phosphorylation and dephosphorylation of target proteins, have been described to be key elements in ROS-mediated signaling events [3–6]. The major mechanism by which these proteins are modified by oxidation involves the presence of key redox-sensitive cysteine residues [7].

The goal of this review is to discuss the mechanisms by which protein kinase Cs (PKCs) can be modulated by ROS and how these processes are involved in the cellular response. The review will focus on the structural mechanism of action of ROS in the activity of these enzymes, how ROS interact with their target molecules, the regulation of these enzymes by oxidants, and finally the major consequences of this tightly controlled mechanism on cell signaling.

## 2. The Protein Kinase C Family

**Figure 1:**
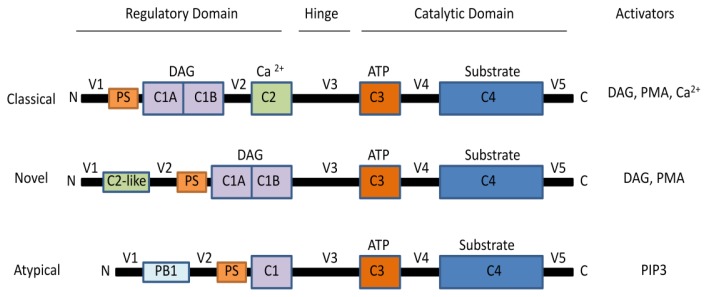
Schematic sequence of protein kinase C (PKC) isozymes indicating the domain structure of the PKC subfamilies and their respective activators.

The PKC family is composed of serine/threonine protein kinases that are involved in a variety of pathways that regulate cell growth, differentiation, apoptosis, transformation and tumorigenicity. Most cells express more than one isoform, and each type of PKC mediates different cellular events [8,9]. The various PKC isoforms consist of NH_2_-terminal regulatory domains and COOH-terminal catalytic domains [10]. They also have in common the pseudosubstrate site (PS), which keeps the protein in its inactive form [11]. However, they differ in their structure, cofactor requirement and substrate specificity [11]. Thus, the 10 members of the PKC family have been divided into three major groups: the classical PKCs (cPKCs), including the α, βI, βII, and γ isoforms; the novel PKCs (nPKCs), including the θ, η, ɛ, δ isoforms; and the atypical PKCs (aPKCs), including the ζ and ι/λ isoforms [12–14]. The cPKC subfamily members possess conserved (C1–C4) and variable (V1–V5) regions, which are presented in Figure 1 [11,12]. They require calcium, phosphatidylserine and diacylglycerol (DAG) or phorbol esters for activation. The nPKCs differ from cPKCs in that they lack the C2 homologous domain and do not require calcium for activation. Finally, aPKCs lack both the C2 and half of the C1 homologous domains, thus rendering them insensitive to DAG, phorbol esters, and calcium [10,15]. Figure 1 depicts the three classes of PKCs and their respective activators. PKCμ and PKCυ isorforms are now classified as members of the DAG receptor protein kinase D (PKD), which is a family of serine/threonine protein kinases classified as a subfamily of the Ca^2+^/calmodulin-dependent kinase (CaMK) superfamily. For a complete review of PKD see [16,17].

### 2.1. Activation of PKCs

PKC isoforms require serine/threonine phosphorylation for their activation. There are three distinct phosphorylation sites in the catalytic domain; the first and rate-limiting phosphorylation step occurs in the activation loop sequence [10,15]. Phosphoinositide-dependent kinase-1 (PDK-1) is the upstream kinase that directly phosphorylates the activation loop of many PKCs [10,15,18]. The activation cascade differs according to the PKC isoform. PDK-1 directs the activation of aPKCs, such as ζ, in a phosphoinositide 3-kinase (PI3K)-dependent manner. In this case, phosphorylation is regulated rather than constitutive. The PI3K pathway produces inositol phospholipids containing an additional phosphate at the third position, which activate PDK-1 and aPKC [19]. However, the phosphorylation at the activation loop of the cPKC and nPKC isozymes does not activate but rather promotes the autophosphorylation of two residues at the *C-*terminus. The first residue has been termed the “turn motif” because it is flanked by a proline residue, and the terminal site is called the “hydrophobic motif” because it is flanked by hydrophobic amino acid residues. The autophosphorylation produces a mature and fully phosphorylated enzyme that is able to respond to lipid second messengers [10,15,18,20].

Phospholipase C (PLC) is an enzyme that, when activated by growth factor receptors, hydrolyzes phosphatidylinositol 4, 5-bisphosphate (PIP_2_) to generate DAG and inositol trisphosphate (IP_3_), which subsequently mobilize intracellular calcium [21]. In the absence of activating cofactors, such as Ca^2+^ and DAG, PKCs are maintained in an inactive conformation by binding of the PS to the substrate-binding cavity [11,20]. The cPKCs are activated by both DAG, which binds to the C1 domain, and Ca^2+^, which binds to the C2 domain. After Ca^2+^ signaling, the binding of DAG to the C1 domain increases the affinity of PKCs for membrane lipids, inducing conformational changes that lead to a catalytically competent form. Phorbol esters bind to the same site as DAG and mimic its activation [11,22,23]. Novel PKCs are independent of Ca^2+^ and activated directly by DAG [11]. Recent studies indicate the existence of another model in which cellular stimulation results in inducible phosphorylation at some of the three sites in PKCs [24–26]. Generally, activation is characterized by the translocation of the PKC from the cytosol to the plasma membrane [27]. However, the isoforms have different subcellular localizations, and this simple model cannot be applied for all of them.

### 2.2. PKC Receptors and Localization of Isoenzymes

PKC isoforms are differentially distributed not only with respect to tissue but also in terms of subcellular localization, suggesting that each isoform could have specific functions [10,11]. Intracellular receptors for inactivated kinases (RICKs) and intracellular receptors for activated kinases (RACKs) are involved in the specific translocation of PKCs from the cytosol to different compartments [28,29]. RICKs and RACKs are proteins that bind PKCs in an isozyme-specific and saturable manner, and any PKC activator should induce the release of PKC from RICK [29]. The interaction of PKCs with RACKs is mediated through the PKC regulatory domain. Moreover, RACKs have been shown to be responsible for the different cellular distribution of the same isoform in different cell types [11].

The α, ζ and δ PKC isoforms are expressed in all tissues [28], while other isoforms are expressed in a tissue specific-manner, such as βI in the spleen; βII in the spleen and brain; η in keratinocytes; and θ in skeletal muscle, T cells and epidermis [28,30]. Atypical PKCι/λ can be found in the testis and insulin-secreting cells [28]. Subcellular localization varies according to organism, tissue, and stimulation. Different diets can also affect the expression and localization of PKC isoforms [10,19,28,31–33].

## 3. Regulation of Protein Kinase C by Reactive Oxygen Species (ROS)

Reactive oxygen species (ROS) are produced in the natural course of metabolism. They can originate from different sources, including the mitochondrial electron transport chain, xanthine oxidase, myeloperoxidase, nicotinamide adenine dinucleotide phosphate (NADPH) oxidases (Nox enzymes), and lipoxygenase [3,25]. Nox enzymes and lipoxygenase are responsible for the production of ROS in response to hormones, growth factors and cytokines [34–36]. Among these oxygen metabolites are superoxide anions (O_2_^−^), hydrogen peroxide (H_2_O_2_) and hydroxyl radicals (·OH) [26]. These species are not all equally reactive with their prospective targets. Many of them have very short half-lives, leading to little relevance in terms of signaling. For example, the ^•^OH is the most unstable radical, reflecting its limited ability to transmit signals across any significant distance. In addition, O_2_^−^ and H_2_O_2_ can be considered more stable species, and because of this feature, they may be the most favorable ROS to operate as signaling molecules [37]. ROS were once considered a dangerous product of cellular metabolism because, in high concentrations, they deleteriously affect DNA, lipids and proteins. Currently, these reactant molecules, in lower doses, are thought to act as important mediators of cell growth, adhesion, differentiation, senescence, and apoptosis by modifying key elements in protein regulatory sites [3,25].

**Figure 2:**
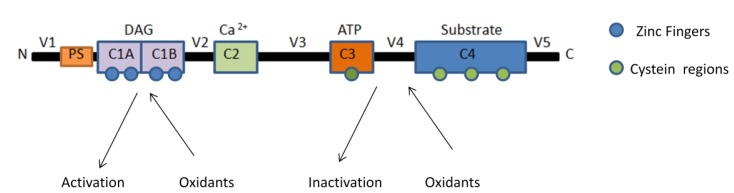
Schematic representation of susceptible sites to oxidation of PKCs.

In the PKC structure, two pairs of zinc fingers are found within the regulatory domain. They are sites of DAG and phorbol ester binding. Each zinc finger is formed by a structure that is composed of six cysteine residues and two zinc atoms, as presented in Figure 2. The high levels of cysteine residues render the regulatory domain susceptible to redox regulation [11,23]. The oxidants destroy the zinc finger conformation [38], and the autoinhibition is relieved, resulting in a PKC form that is catalytically active in the absence of Ca^2+^ or phospholipids. Cysteine residues are also found in the *C-*terminal catalytic domain, but they are uncoordinated in a different manner to those in the regulatory domain. Redox modifications at the *C-*terminal catalytic domain lead to the inactivation of the kinase due to the loss of the free sulfhydryls required for its catalytic activity. Furthermore, the intracellular redox state has been shown to affect the distribution of the PKC isoforms [11,23]. Because all PKC isoforms possess zinc fingers and high concentration of cysteine residues located in the regulatory domain, as well as free sulfhydryls in the catalytic site, it seems that the regulation by oxidation is a common feature for this family. However, this signaling mechanism may vary according to the distinct isoform and different cell [11]. The aPKCs, for example, lack one of the two cysteine-rich zinc-finger regions in the regulatory domain [39]. This characteristic can render aPKCs to a distinct susceptibility of oxidative stress. In mouse embryonic fibroblasts, oxidative stress triggers translocation of PKCα, β, δ, and ɛ isoforms from the cytosol to the plasma membrane. Nevertheless, under the same conditions, PKCζ translocates to the nucleus [11]. PKCδ selectively regulates the activation of the inducible nuclear factor κB (NF-κB) in response to oxidative stress. The activation of the transcription factor NF-κB is often observed in cells exposed to oxidative stress [40]. In HeLa cells treated with exogenous H_2_O_2_ this signaling pathway was shown to be dependent of the activation of PKD by two coordinated signaling events: the phosphorylation of Tyr463 mediated by the Src-Abl signaling pathway, which then facilitates the second step, the phosphorylation of the PKD activation loop of Ser738/Ser742 by the Src-PKCδ pathway, leading to an increase in cell survival exposed to oxidative stress [41,42]. This coordinated mechanism seems to be specifically in response to oxidative stress, and relies on the activation of PKCδ and not of other PKC isoforms [41]. Later the same group showed that the resveratrol-dependent inhibition of PKD Ser738/Ser742 phosphorylation by PKCδ was able to block the activation of NF-κB in response to oxidative stress. Resveratrol inhibited NFκB activation by avoiding PKD association with IKK complex [40]. The specificity by which ROS may activate different PKC isoforms is not well established, but the localization and the intensity of ROS generation could contribute to the explanation of such different responses.

## 4. Effect of Mitochondrial ROS Generation on PKC Activity

In addition to hormones and growth factors, ROS are currently recognized as initiating factors for signaling cascades in the cell, as they are involved in numerous physiological signaling pathways and cellular functions. Thus, sites of ROS production within the cell, such as mitochondria, or enzymes that are capable of one-electron transfer to O_2_, such as Nox enzymes, xanthine oxidase and cytochrome P-450, play important roles in cellular metabolism and signaling [34–36].

The main superoxide producer in the cell is the respiratory chain, and the primary factor governing mitochondrial ROS generation is the redox state of the respiratory chain [43,44]. In fact, two of the respiratory chain complexes (I and III) have long been recognized for their involvement in O_2_^−^ production. With respect to complex III, two components were proposed to be auto-oxidizable electron donors to oxygen: semiubiquinone and reduced cytochrome b. The other site in the respiratory chain that is involved in O_2_^−^ formation is complex I. In this large multi-subunit complex, electron transfer functions at near equilibrium, and thus, O_2_^−^ production may be linked to both forward electron transport and reverse electron transport. For a complete review, see Rigoulet *et al.* 2011 [45].

PKCs exist in an inactive form and are activated depending on their subcellular redistribution and the availability of the appropriate substrates after a distinct stimulus [46,47]. Currently, much evidence supports the direct activation of different PKC isoforms by ROS generation [23,48]. The signaling pathway triggered upon PKC activation by ROS depends on the specific isoform, cell type, and the site of ROS generation. Thus, depending on the particular condition, this mechanism can be involved in either cell protection or death.

The presence of PKCs in the mitochondria has long been known, but their functions are not well defined [47,49]. The activation of PKCδ with phorbol myristate acetate (PMA) or H_2_O_2_ stimulates its accumulation in the mitochondria, and this fact can be related to apoptosis in different cell types [50–52]. On the other hand, accumulation of PKCɛ in the mitochondria was appointed a cardioprotective function [53].

In human melanoma cells, mitochondrial ROS were found to have a cytoprotective effect in cells treated with the chemotherapeutic silibinin. In a study performed by Jiang and colleagues [54], silibinin induced ROS generation in these cancer cells, with O_2_^−^ being the major species responsible for the activation of PLC-dependent PKCγ, which is part of a pro-survival pathway to protect the cells from the cytotoxicity of silibinin [54].

PKC activation is also involved in cell damage induced by hyperglycemia. Hyperglycemia results in a loss of antioxidant reducing equivalents, which culminates in an overproduction of O_2_^−^ and Sorbitol, the product resulting from the enzymatic conversion of glucose, can produce fructose by the action of sorbitol dehydrogenase. This reaction increases the ratio of NADH/NAD^+^, enhancing the oxidized form of triose phosphates with *de novo* synthesis of DAG. DAG can then activate several PKC isoforms, which can lead to various clinical complications in diabetes [55,56]. In bovine vascular endothelial cells, the inhibition of mitochondrial metabolism or the overexpression of uncoupling protein-1 (UCP1) or superoxide dismutase (SOD), the enzyme responsible for the dismutation of O_2_^−^ to H_2_O_2_, results in a decrease in mitochondrial ROS production, which prevents PKC activation and sorbitol accumulation [57].

Mitochondrial ROS also have an important role in pulmonary vasoconstriction induced by hypoxic conditions. In artery smooth muscle cells that were submitted to an acute hypoxic period, an increase in mitochondrial ROS production was shown, followed by the activation of total PKC and PKCɛ. The experiments performed by Rathore and colleagues [58] provided support for the specificity of mitochondrial ROS-induced signal transduction pathways in vasoconstriction induced by hypoxia. The activation of PKCs in response to hypoxic conditions in pulmonary artery smooth muscle cells is remarkable because different responses were seen in mesenteric arteries under the same conditions. Moreover, the external addition of H_2_O_2_ was able to mimic hypoxic responses in normoxic conditions, leading to an increase in PKCɛ activity in pulmonary arteries. Interestingly, H_2_O_2_ also significantly activated PKCɛ in mesenteric arteries although not under hypoxic conditions [58]. These experiments highlight the importance of H_2_O_2_ as a second messenger, but the effectiveness of the signal triggered by this molecule may also be related to the site of its production, in this case, the mitochondria. In hepatocytes, mitochondrial ROS generation after cell exposure to sodium arsenite (NaAsO_2_) was shown to induce PKCδ activation, which in turn activates c-Jun *N-*terminal kinases (JNK) leading to the progression of apoptosis. On the other hand, in the same study, the authors showed that treatment of hepatocytes with taurine was able to inhibit PKCδ and JNK activation, thus avoiding arsenic-induced ROS generation in the liver, preventing apoptosis [59]. In contrast, PKCδ is involved in γ-radiation activated signaling in splenic lymphocytes with a cytoprotective effect. In this case, radiotherapy treatment leaded to a decrease in the activities of antioxidant enzymes and an increase in cell oxidative damage. This effect was followed by an increase in PKCδ activation and degradation of IκBα during the earlier period of treatment. IκBα after degradation is known to release NF-κB, which acts in the nucleus as a transcription factor for many anti-apoptotic genes, thus indicating that the cell could be entering into a cytoprotective pathway. In this study, pretreatment of irradiated cells with curcumin-copper complex was shown to be effective in avoiding radiation-induced damage in these cells [60].

ROS-induced PKC activation also has an important function in the cardioprotective signaling cascade. Sevoflurane, an anesthetic with the ability to protect the myocardium from ischemia and reperfusion injury, triggers a protective signal transduction cascade [61]. The preconditioning elicited by sevoflurane involves the translocation of PKCα towards the mitochondria, which is induced by an increase in ROS production [62]. The fundamental role of PKCα in this cardioprotective effect is still unclear, but the authors suggest the participation of this enzyme in an anti-apoptotic signaling cascade [62]. On the other hand, PKCs can be involved in apoptosis induction through mitochondrial ROS generation. Treatment of head and neck squamous carcinoma cells (HNSCC) with *N-*(4-hydroxyphenyl)retinamide (4HPR), a synthetic retinoid effective in cancer chemoprevention and therapy was shown to increase mitochondrial ROS generation, which in turns activates PKC and MAPK kinases (MKK4,MKK3/6). Downstream of PKC activation is the activation of p38 and ERK, resulting in an enhancement of apoptosis via caspase 9 and caspase 3 pathway [63].

In addition to interactions between receptors and ligands, other events are involved in mitochondrial ROS-induced PKC activation. Several studies implicate that mechanical stretch increases ROS production in endothelial cells [64–66]. In bovine pulmonary artery endothelial cells, strain-induced perturbation of the plasma membrane is involved in the release of ROS to the cytosol, thus activating downstream effectors involved in the mechanotransduction signaling pathway. These signals involve the participation of the intact actin cytoskeleton and the phosphorylation of focal adhesion kinase (FAK) by PKCα. In this way, PKC activation is preceded by mitochondrial ROS production in stretch-induced endothelial cells [67].

## 5. PKC Phosphorylates Nox Subunits for ROS Generation

Nox enzyme is a complex multi-subunit enzyme that uses NADPH as a substrate to convert molecular oxygen into ROS (mainly O_2_^−^ with secondary production of H_2_O_2_) in a regulated manner in response to different stimuli, including growth factors, cytokines and calcium signals [68,69]. The prototypical Nox enzyme from phagocytic cells is divided into two parts: a membrane bound component, the flavocytochrome *b*_558_, which is composed of two subunits, a large glycosylated gp91phox that forms the catalytic subunit and the smaller regulatory subunit p22pho*x*, and a cytosolic component formed by four major proteins, the regulatory subunits p47phox, p67phox, and p40phox, and the small GTPase RAC [70–73]. To date, seven mammalian isoforms are known (Nox1-5, Duox1, 2) to be expressed in a wide variety of tissues, such as vascular smooth muscle, kidney, spleen, cerebrum, lungs, colon, ovary and others [74]. When cells are stimulated, the cytosolic components migrate almost instantly to the membrane, where they assemble with the flavocytochrome *b*_558_ to form the active enzyme, a process that is tightly regulated by protein-protein interactions and phosphorylation of the p47phox subunit [68,70–73,75].

PKC isoforms are upstream regulators of Nox. The activity of these enzymes is important for the assembly and activation of the Nox1-3 isoforms, which require the phosphorylation-dependent assembly of several cytosolic subunits to be catalytically active; this includes p47phox, which is the best studied of the phosphorylated Nox enzyme components [76]. The *C-*terminal sequence of p47phox has a basic charge, is rich in serine and arginine residues and has at least one proline-rich region (PRR) (amino acids 363–368) [71,77]. In resting cells, p47phox is not phosphorylated and possesses a highly basic charge (pI > 9). Upon phosphorylation, its pI shifts to the acidic range, giving rise to several phosphorylated isoforms, which correspond to different phosphorylated states [71,78]. Different PKC isoforms in various tissues have been described to be efficient in the phosphorylation of the p47phox subunit, as summarized in Table 1. On the other hand, both Nox4 and Nox5 do not require cytosolic subunits for their activation. Nox4 is a constitutively active enzyme and is bound to the integral membrane protein p22phox, whereas Nox5 is primarily regulated by calcium levels [79,80].

PKC is not the only protein kinase able to phosphorylate p47phox. PKA [95,96], MAPK ERK1/2 and p38MAPK [97,98], protein casein kinase 2 (CKII) [99], AKT [100,101], p21-activated kinase (PAK) [102], a phosphatidic acid-activated kinase [103] and src kinase [104] have also been shown to phosphorylate this subunit [71].

A growing body of research has shown that the activation of the PKC/Nox signaling complex regulates ROS levels and is involved in various pathophysiological conditions, including neurodegenerative disorders [105,106], human cardiovascular disease, such as atherosclerosis [95,107–111], hypertension [112,113], renal damage [91], diabetes [114–117], and cancer [118].

PKC has been shown to activate O_2_^−^ generation by Nox enzymes in several types of cells, including phagocytes [46,71], cardiomyocytes [100], aortic endothelial cells [107], platelets [114], and renal mesangial cells [119].

The interaction between ligand and receptor is crucial to trigger the signaling pathways that involve the PKC and Nox enzymes. The response is different in each cell and involves distinct PKC and Nox isoforms. In vascular smooth muscle cells (VSMC), for example, three isoforms of Nox enzymes are present, Nox1, Nox2, and Nox4. They are thought to have different functions and subcellular localization. It seems that Nox1, when activated, is present on the cell surface and co-localizes with caveolins, while Nox2 can be located intracellularly or in the cell membrane. Nox4 is localized in focal adhesions and in nuclei [120–122]. In this model, Nox4 plays an important role in the constitutive production of ROS, while Nox1 would be necessary during pathological development [122–124]. Recently studies showed that Nox1 expression is involved in the hypertrophy of VSMCs induced by aldosterone and prostaglandin F2α. In both cases, the up-regulation of Nox1 may occur through the activation of PKCδ and the activating transcription factor 1 (ATF1) [122,125,126].

PKC can also activate Nox2. In human monocytes and murine macrophages PI3K and PKC pathways are involved in Nox2 stimulation in response to insulin. This mechanism triggers ERK1/2, p38MAPK and NFκB, which culminates in the activation of monocytes and proliferation of macrophages [127]. PKC-dependent Nox2 activation was also shown in coronary artery disease. Despite the concomitant expression of Nox2 and Nox4 in these arteries, Nox2 represents the main source of superoxide production in the disease, and this was related in part by an increase in monocyte/macrophage infiltration, which reflects the presence of inflammatory cells. On the other hand, Nox4 seems to be related to smooth muscles cells or myofibroblasts and is independent of the pathological condition [128].

Several studies demonstrate that Nox4 is inducible and can generate H_2_O_2_ in response to various hormone stimuli. Generation of H_2_O_2_ by Nox4 after insulin stimulation of differentiated adipocytes was shown to be important in both early and late events in insulin signal transduction, which includes the activation of insulin receptor substrate-1 (IRS-1) tyrosine phosphorylation, and the activation of downstream serine kinases and glucose uptake [129]. Similar results were found in preadipocytes, where Nox4 acts indirectly to facilitate the tyrosine phosphorylation of IRS-1, stimuli required for the insulin-induced differentiation of these cells [130]. Nox4 was also stimulated by H_2_O_2_ and diacylglycerol, through a mechanism dependent of phospholipase A_2,_ but apparently independent of PKC activity. The arachidonic acid generated in this system by phospholipase A_2_ is responsible for the oxidase activation and the increase of intracellular ROS production [131], similar results were found in mesangial cells [132].

It has been well established that angiotensin II exerts important intra–cellular signaling through ROS generation in many physiological and pathological conditions [133]. In many published works, angiotensin II has been shown to activate the Nox enzyme in a PKC-dependent manner [91,109,134–138]. Apoptosis of cardiac cells mediated by alcohol is dependent on the angiotensin II interaction with the angiotensin II type 1 (AT1) receptor, which subsequently activates PKCβI to phosphorylate and activate the Nox enzyme. Because high amounts of superoxide are generated in this process, this mechanism has also been related to alcoholic cardiomyopathy [134]. Angiotensin II also increased the basal and the NADPH stimulated superoxide production in coronary arteries, and this was found to be modulated by PKC activity [128]. In addition, angiotensin II stimulates the epithelial Na^+^-channel in the rat cortical collecting duct through the activation of Nox-dependent PKC phosphorylation [135]. In the renal system, this receptor agonist has a stimulatory effect in a cross talk mechanism between the connecting tubule in the renal cortex and the afferent arteriole, which is mediated by O_2_^−^ that is generated primarily by PKC-dependent Nox activation [132]. In physiological conditions, angiotensin II also regulates O_2_^−^ generation in the thick ascending limb of the renal medulla through the activation of PKCα and the stimulation of NADPH oxidase [81]. This mechanism is also associated with an increase in sodium absorption in this renal structure [136]. Similar results were obtained for skeletal muscle [137] and monocytes, in which increased ROS generation by angiotensin II caused an increase in the level of interaction between human monocytes and the extracellular matrix by favoring adhesion to laminin-1 [108]. In myocytes, White and collaborators showed that this angiotensin II-dependent activation of PKC/Nox inhibits the Na^+^–K^+^ pump [84]. Conversely, at the same time that angiotensin II triggers PKC-dependent Nox enzyme activation and ROS generation, it also stimulates the activity of antioxidant enzymes like catalase, SOD, and glutathione peroxidase (GPx) in the rat hypothalamus, a counteraction mechanism that exerts local control on ROS levels [138].

Angiotensin II can also act as key neuromodulator in central autonomic nuclei [139,140]. Vagal afferent neurons, in the nucleus of the solitary tract, play a major role in cardiovascular regulation and are a target of angiotensin II through activation of the AT1 receptor. In these cells intracellular Ca^2+^ and PKC activation are critical for AT1 receptor-induced Nox2 activation and ROS production. The authors suggest that this mechanism could play a role in the vascular dysregulation mediated by the central autonomic system [141].

Phorbol esters, such as 12-*O*-tetradecanoylphorbol-13-acetate (TPA) or PMA, are also involved in PKC-dependent Nox activation. In human lung adenocarcinoma cells, the tumor-promoting TPA and the tumor necrosis factor-alpha (TNF-α) were implicated in the stimulation of the expression of the mitochondrial manganese-dependent isoform of SOD (MnSOD) mediated by the Nox enzyme pathway. This stimulation is in part regulated by PKCα, which phosphorylates a transcription factor of cAMP-responsive element-binding protein, or by the PKCɛ pathway, which culminates with the activation of forkhead transcription factor 3 (FOXO3) [142]. In a recent study, Kamiya and colleagues showed that the reduction of extracellular SOD and cytosolic copper/zinc SOD (Cu/ZnSOD) is dependent on the PKC/Nox complex during the TPA-induced monocytic differentiation of U937 cells, a leukemic cell line. Moreover, the nuclear factor kappa B (NF-κB) is involved only in the reduction of Cu/ZnSOD [143], suggesting differential regulation for both isoforms. In human umbilical vein endothelial cells, TNF-α was also shown to increase ROS generation via the PKCβII-dependent activation of vascular Nox enzyme. This mechanism has been suggested to be involved with apoptosis in these cells, but it occurs independently of ROS generation [144]. PMA influences the phosphorylation of Nox5 through the activation of the MAPK/ERK1/2 pathway, leading to an increase in ROS generation in fibroblasts [145] in a calcium-independent manner [146]. Although Nox5 requires calcium for its activation, the specific phosphorylation of serine/threonine residues in Nox5 affects the calcium sensitivity of Nox5, thus permitting its activation without a change in calcium levels in the cell [80,146,147]. In bovine coronary arteries, PKC is activated by phorbol 12,13-dibutyrate (PDBu). PDBu increases superoxide generation by Nox2 through both p47phox and peroxide-dependent Src activation. The authors suggest that Nox2 contributes to vascular contractile mechanisms through PKC-dependent activation [148].

Several works also highlight the participation of formyl peptide formyl-methionyl-leucyl-phenylalanine (fMLP) in the activation of PKC and Nox enzyme. This participation includes superoxide generation, which in turn stimulates leukocytes, phagocytic cells, and neutrophils [46,72,84,91,149,150].

Although most biological functions of PKC have been recognized to occur at the plasma membrane or in the cytoplasm, several studies have pointed out the role of PKCs in nuclear functions [39]. Almost all the isoforms of PKC have been identified at nuclear level, except for the PKCμ isoform [39,151]. The distinct functions of PKCs in the nucleus may be a consequence of either, the activity of resident isoforms of the enzyme or the translocation of PKC from cytoplasm to the nucleus, which may occur in certain conditions and in a different cellular system, as a result of nuclear lipid signaling [39]. It seems that DAG is the driving force to attract cPKCs to the nucleus [39,152–155], while for nPKCs and aPKCs there has been no specific recognized lipid messenger for this function until now. However, Phosphatidylinositol (3,4,5) trisphosphate (PtdIns(3,4,5)P3), one of the products of PI3K family members, has been proposed to act as the driving force for the nuclear translocation of the aPKC isoform PKC-ζ [39,156]. This PKC-ζ translocation through PtdIns(3,4,5)P3 signaling has been shown in nerve growth factor (NGF)-treated rat pheochromocytoma PC-12 cells [157,158], and in rat epatocytes treated with C2-ceramid [159].

The nuclear presence of PKC suggests an involvement of these enzymes in the regulation of DNA replication, RNA synthesis and processing, gene expression, transport between nucleus and cytoplasm, and chromatin structure [39]. Moreover, nuclear PKCs have been implicated in cell proliferation, differentiation, apoptosis, and cardiovascular diseases [39,160]. For a complete review of nuclear PKCs and their function in cells see [39,151,161].

Beside PKCs, Nox enzymes subunits have been described to be localized in subcellular compartments of different cells [162]. This includes the presence of Nox1 and Nox4 in endoplasmic reticulum [79,163], Nox2 in perinuclear compartments [164], and Nox4 at the nucleus of multiple kinds of cells, such as endothelial cells, VSMCs, and hepatic cells [121,165,166]. Nox4 is considered the predominant NADPH isozyme in the kidney with prevalent expression in the proximal tubule [167,168]. Regarding these concepts, recent data show that angiotensin II can induce a nuclear Nox4-dependent generation of ROS, through PI3K and PKC activation in isolated nuclei of cortical cells of kidney [169].

## 6. PKC-Induced ROS Generation

PKC also has been shown to promote the production of endogenous ROS to induce a positive feedback loop [48]. The β isoform of PKC induces ROS generation through mitochondrial damage [170]. The enzyme has two major splice variants, -βI and -βII, which result from alternative splicing and differ in their carboxyl-terminus and subcellular localization [171]. In melanoma, the reduced expression of the variant PKCβII decreases ROS generation and promotes the survival and growth of melanoma cells under oxidative stress conditions [172]. In chondrocytes that have lost their normal extracellular matrix and growth factor survival signals, the activation of PKCβI results in the production of mitochondrial ROS, which is required for a signal that mediates both apoptosis and necrosis. Notwithstanding, in this same study, PKCδ activity was determined to be necessary for chondrocyte survival [48]. The PKCβ isoform is responsible for the activation/phosphorylation of the mitochondrial p66^shc^ protein [173]. This protein is an alternatively spliced isoform of a growth factor adapter. When this isoform is activated in mitochondria, it can bind to cytochrome c, acting as an oxidoreductase and ROS-generating system and subsequently promoting organelle dysfunction and cell death [174]. In mouse embryonic fibroblasts, inhibition of PKCβ leads to the inhibition of mitochondrial p66^shc^ phosphorylation, preserving mitochondrial morphology and reducing apoptosis in cells under oxidative stress [170].

In myeloid leukemia cells, ROS are not necessary for the translocation of PKCβII from the cytosol to the cell membrane. However, PKCβII was shown to be essential for ROS production induced by the PKC activator TPA. This ROS-dependent mechanism is associated with the stress-activated protein kinase pathway (SAPK). SAPK is associated with the molecular response of myeloid leukemia cells to TPA, through the phosphorylation and activation of c-jun, activating transcription factor 2 (ATF2) and Elk-1, contributing to the induction of the c-jun and Erg-1 early response genes involved in this signaling process [175]. The PKCɛ and PKCζ isoforms are also involved in mitochondrial ROS production. The neuroprotective effect of the sphingolipid C2-ceramide involves the participation of both isoforms, which promote an increase in the formation of mitochondrial ROS and a controlled opening of the mitochondrial permeability transition pore. In ischemia, these events prevent mitochondrial calcium overload, leading to a protective effect and the prevention of cell death during recovery of normal cell function [176]. In skeletal myocytes and glioma cells, PKCα is translocated to the mitochondria after activation by PMA, resulting in mitochondrial dysfunction through a decrease in complex I activity and an enhancement of ROS generation [47].

## 7. Conclusions

**Figure 3:**
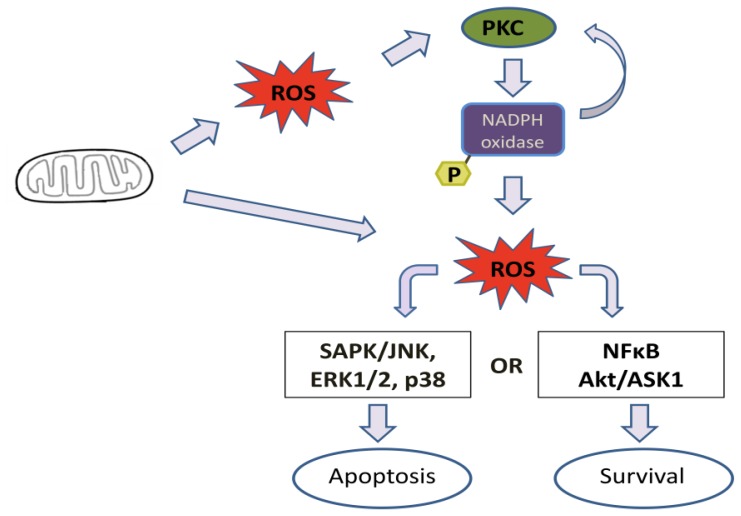
General mechanism of cell signaling activation through mitochondria/PKC/nicotinamide adenine dinucleotide phosphate (NADPH) oxidase. In pathological conditions, a mitochondrial dysfunction could lead to an increase in reactive oxygen species (ROS) generation. ROS can directly trigger cell signaling or activate different PKC isoforms, depending on cell type and stimulation. Activated PKC stimulates NADPH oxidase, which generates ROS. These ROS could activate another group or the same group of PKCs in a feedback mechanism or induce cell signaling. Depending on the stimulus, it could lead to cell survival or death.

Because different PKC isoforms are present in a variety of cells and are involved in a number of signaling pathways, it is difficult to ascribe a common ROS-dependent mechanism for all PKC isotypes. However, it seems that under pathological conditions, a general signaling mechanism is triggered in mitochondrial dysfunction. In this context, an increase in ROS production by mitochondria activates local PKCs, which in turn activate Nox enzymes. Nox enzymes could also activate another group or the same group of PKCs in a feedback mechanism. Consequently, the redox state of the cell becomes imbalanced. Depending on the stimuli, proapoptotic signaling may occur through ROS activation of MAPK, such as SAPK/JNK, ERK1/2 and p38, or antiapoptotic signals ccould be triggered through activation of the NFκB or Akt/ASK1 pathway [177]. Figure 3 represents a general mechanism of cell signaling activation trough mitochondria/PKC/NADPH oxidase. This mechanism can be different depending on the cell and activation stimulus. More studies are necessary to elucidate the mechanisms that are involved in these signaling pathways.

## Figures and Tables

**Table 1:** Protein kinase C (PKC) isoforms involved in phosphorylation of p47phox subunit.

PKC group	PKC isoform	Cell/tissue type	References
Classical	PKCα	Kidney	[81]
	PKCβ	Leukemic cells; Neutrophils; and Monocytes.	[46,82–85]
Novel	PKCδ	Monocytes; Neuroblastoma; Neutrophils; and Fibroblast.	[75,86–88]
	PKCɛ	Pulmonary artery smooth muscle; and Myocytes.	[89,90]
Atypical	PKCζ	Leukocytes; Neurons; Hippocampus of mice; and Alveolus.	[91–94]
